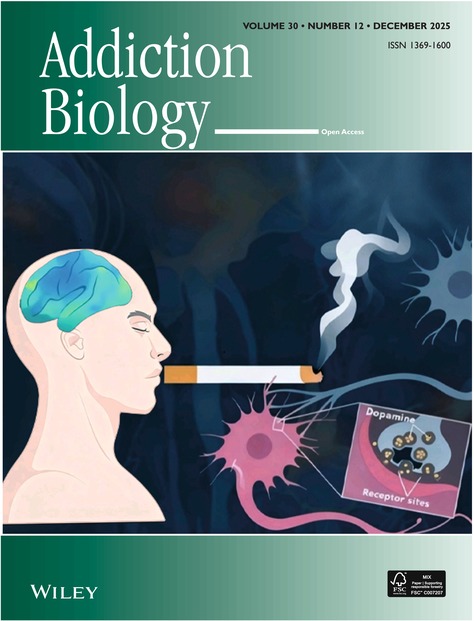# Cover Image

**DOI:** 10.1111/adb.70120

**Published:** 2025-12-22

**Authors:** Hui Zhang, Jiawen Tian, Xinyu Wang, Hongyu Zhang, Longyao Ma, Bohui Mei, Mengzhe Zhang, Qingqing Lv, Yarui Wei, Shaoqiang Han, Yong Zhang

## Abstract

The cover image is based on the article *Functional Connectivity Disruptions in Grey Matter Volume‐Altered Brain Regions Among Male Smokers: A Neuroimaging Study* by Hui Zhang et al., https://doi.org/10.1111/adb.70096.